# Increased dietary fiber is associated with weight loss among Full Plate Living program participants

**DOI:** 10.3389/fnut.2023.1110748

**Published:** 2023-04-17

**Authors:** Rebecca K. Kelly, Janet Calhoun, Amy Hanus, Pamela Payne-Foster, Ron Stout, Bruce W. Sherman

**Affiliations:** ^1^Element Health, Inc, Fairhope, AL, United States; ^2^Ardmore Institute of Health (AIH), Ardmore, OK, United States; ^3^College of Community Health Sciences, University of Alabama, Tuscaloosa, AL, United States; ^4^School of Health and Human Sciences, University of North Carolina at Greensboro, Greensboro, NC, United States

**Keywords:** workplace, nutrition, dietary fiber, plant predominant, weight loss, whole food, intervention

## Abstract

**Introduction:**

Prior studies have demonstrated that an intake of foods rich in dietary fiber is associated with a favorable impact on health status and body weight. However, the association between fiber intake and weight loss has not been well-studied in employer settings. This research aimed to assess the relationship between dietary fiber and weight loss among individuals participating in the Full Plate Living (FPL) program.

**Methods:**

The 16-week plant-predominant fiber-rich eating program was delivered to 72 employers, primarily in the Southwest U.S., over 3 years (2017–2019). Participants received weekly video lessons, FPL materials, and additional online resources. A retrospective analysis of repeated measures was conducted using participant data obtained from 4,477 participants, of which 2,792 (62.5%) reduced body weight. Analysis of variance with *post hoc* analysis was used to assess the statistical significance of the changes between baseline and follow-up measures of dietary fiber intake in each of the food categories, specifically the relationship between changes in individual and combined (composite) daily servings of fruits, vegetables, whole grains, beans, and nuts on body weight measures among three groups at follow-up: those who lost, maintained, or gained weight. Multilevel modeling was used to test the hypothesis that increased intake of fiber was associated with greater weight loss.

**Results:**

The mean weight loss for the weight loss group was 3.28 kg. As compared to the two other groups, the intake of whole fiber-rich foods at follow-up was significantly higher among the weight loss group with fruits (2.45 servings), vegetables (2.99 servings), beans (1.03 servings), and total fiber composites (9.07 servings; *P* < 0.001). A significant increase in servings of grains was also noted (*P* < 0.05). Multilevel modeling demonstrated that a higher total fiber composite (Model 1), as well as higher intakes of either vegetables or fruits (Model 2), resulted in greater weight loss.

**Discussion:**

Our findings indicate that the FPL program can be a part of a lifestyle medicine approach to healthy eating and weight loss. Delivering the program in clinical, community, and workplace settings can increase its reach as an effective and low-cost offering.

## Introduction

The workplace provides employers with opportunities to identify solutions to enhance employee health, wellbeing, and productivity while maintaining or reducing healthcare costs (1–6). Being overweight and obese is linked to increased healthcare expenditures across adults of all ages and body mass index ranges, particularly for individuals with severe obesity (7). Excess body weight is also linked to numerous comorbid and chronic health conditions including type 2 diabetes, heart disease, stroke, and various types of cancer. The Centers for Disease Control and Prevention Obesity (8) reports that the prevalence of obesity among adults living in the United States is 41.9%, of which ~60% of adults in the US are working (9). With the increased prevalence of obesity in the US, recent studies have expanded our understanding of dietary contributors to weight loss. Factors associated with weight loss are multifaceted and include adjustments in dietary intake of kilocalories, macronutrients, and fiber. Previous research suggests that a higher intake of dietary fiber is a predictor of weight loss and compliance with healthy eating when combined with an overall reduction in kilocalorie intake (10–14). Intake of foods high in fiber has also been associated with improved health, quality of life, and reductions in chronic health conditions (11–19).

Full Plate Living (FPL), a multicomponent nutrition education program, was designed for individuals and groups in clinical, workplace, and community settings. The details and benefits of the FPL program have been previously described (20–22). FPL's simplified healthy eating approach emphasizes small-step habit adoption to include the consumption of plant predominant foods high in fiber, such as fruits, vegetables, beans and legumes, cooked whole grains, and nuts (21). The focus of the FPL plant-predominant nutrition program is to gradually consume 75% of a meal from nutrient-dense, low-calorie foods that are high in fiber, or ~40 g of fiber per day, over time. FPL also emphasizes physical movement, consumption of water, and awareness of satiety cues.

The beneficial impact of FPL on improving eating behaviors and health values has been demonstrated in both community and employer settings (20, 23–25). A recent FPL analysis demonstrated significant improvements in eating behaviors and health status including increased consumption of fiber-rich foods of fruits, vegetables, whole grains, beans, and nuts; improvement in perceived health and energy values; increased confidence values of losing weight and choosing healthy foods, and overall weight loss (20). However, research gaps exist regarding our understanding of the relationship between weight modification and adherence to the FPL plant-predominant eating approach in the workplace.

This study aimed to assess the relationship between servings of whole-food plant-based foods high in fiber and the impact on body weight among employees in a workplace wellness program using the FPL program. More specifically, the present study is attempting to answer the following three questions:

(1) Does participation in the FPL program result in weight modification?
(2) As a result of participating in the FPL program, does total fiber intake or specific high-fiber foods play a role in weight loss; and
(3) To what extent does the FPL program yield differences across the following three groups: weight loss (WL), weight neutral (WN), and weight gain (WG)?

## Methods

### Study design

The study design, procedures, and intervention have been previously described in detail (20). The 16-week FPL program consisted of individuals receiving weekly online educational videos, FPL program curriculum materials, and online resources to include recipes and meal planning tips. A repeated measures study design was conducted using participant data from the FPL. A 20-question, self-reported confidential online health assessment was completed at both baseline and follow-up (within 3 weeks of program completion) and included questions about eating behaviors, perceived values of energy and health, and confidence values for healthy eating and weight loss. Daily servings of fiber-rich food, including fruits, vegetables, beans, grains, and nuts, were recorded by participants in a food diary. Because this study was a retrospective, *post hoc* analysis of de-identified data, IRB approval for the study was not obtained.

### Subjects

The program was delivered through 72 employers located primarily in the Southwest U.S. from 2017 to 2019. Employee participants were recruited through their organization's wellness program. A total of 6,820 individuals were enrolled in the FPL program, of which 4,477 were included in the analysis as they completed the baseline and follow-up health questionnaires.

### Measures

Measures of data-specific variables were previously described (20), including demographic characteristics, food intake, self-perceived health and energy values, confidence scores, and body weight.

#### Demographic characteristics

Available demographic details were limited to age and gender. *Gender* was coded as male or female.

#### Food intake

Food intake was self-reported by asking “How many actual servings of food do you consume each day over the last 2 weeks for each of the following food categories, such as fruits, vegetables, beans, whole grains, nuts?”

#### Self-perceived health

Responses were coded into five categories: excellent, very good, good, fair, and poor.

#### Self-perceived energy

The self-perceived energy question was assessed on a 5-point scale by asking “How would you describe your energy level in the past month?” Responses were coded into five categories: very high energy, high energy, moderate energy, low energy, and no energy.

#### Confidence scores

Participants recorded their confidence for each item on a 10-point scale, ranging from 0 (no confidence) to 10 (complete confidence).

Additional measures were evaluated for this study to include body weight, fiber composite, and overall water intake.

#### Body weight categories

Body weight was self-reported and provided a comparative measure of pre- and post-program change. Participants were divided into three groups based on their baseline and follow-up program weight change: WL, WN, and WG. Individuals recorded their weight to the nearest pound measurement. Participants who lost >1 pound or 0.45 kg of body weight were included in the WL group. Participants who maintained their weight, <1 pound or 0.45 kg, of body weight change were included in the WN group. Individuals who gained more than one pound or 0.45 kg were included in the WG category.

#### Fiber composite

A high-fiber food composite was developed by adding the total number of daily servings of fiber-rich foods.

#### Water intake

Water was assessed based on the number of cups of water consumed per day.

### Analyses

Analysis of variance with *post hoc* analysis was used to assess the statistical significance of the changes between baseline and follow-up measures of dietary fiber intake in each of the food categories, specifically the relationship between changes in individual and combined (composite) servings of fruits, vegetables, grains, beans, and nuts on body weight measures among the three weight groups. The WL group was used as the reference with a comparison to both WN and WG values for the *post hoc* measures.

Multilevel modeling was used to test the hypothesis that increased fiber intake was associated with lower body weight at follow-up (i.e., weight loss). Random-intercept models were estimated with repeated measures nested in subjects. Two models were estimated. Model 1 had fiber composite as the independent variable of main interest. In comparison, Model 2 examined individual high-fiber food categories including vegetables, fruits, nuts, beans, and whole grains. Included as time-varying covariates were water intake, and a variable (time), to capture secular trends. Time-varying variables were person-mean centered to estimate their within-subject association with body weight. With two repeated measures, person-mean centering was equivalent to a change score. Age and gender were included as time-invariant covariates.

## Results

The program included 6,820 program participants; of which, 4,477 individuals completed baseline and program self-reported questionnaires; therefore included in the program analysis.

Table 1 presents descriptive statistics for baseline demographic characteristics for all participants (*n* = 4,447) including subgroups based on body weight category. The average age for all participants was 45.7 (SD + 11.7). Consistent with similar nutrition and weight management studies demonstrating that women participate at rates three times greater than men (26, 27), the majority of study participants were women (87.0%). Of the three weight groups, the WL category was the largest, at 62.5% (*n* = 2,792), with an additional 22.0% gaining weight (*n* = 988) and 15.5% maintaining their weight (*n* = 697). The mean body weight for all female participants was 86.3 kg (SD + 21.8), while the mean body weight for male participants was 101.3 kg (SD + 22.0).

**Table 1:** Baseline demographic characteristics of participants by weight loss category.

**Variable**	**Total**	**Weight loss (WL)**	**Weight neutral (WN)**	**Weight gain (WG)**
Number	4,477	2,792	697	988
Percentage of total, %	100	62.5	15.5	22.0
Female, %	87.0	86.9	85.4	88.5
Male, %	13.0	13.1	14.6	11.5
Age, years *M* ±*SD*	45.7 ± 11.7	46.0 ± 11.8	45.2 ± 11.8	45.1 ± 11.5
**Baseline body weight, kg**				
Female	86.3 ± 21.8	87.7 ± 22.1	83.0 ± 20.9	84.7 ± 21
Male	101.3 ± 22.0	102.1 ± 20.9	101.8 ± 25.3	98.5 ± 22.3

Data are drawn from the Full Plate Living participant survey data.

Baseline and follow-up measures of participant eating behaviors, health measures, and confidence levels among all three weight groups are presented in Table 2. Significant weight loss was demonstrated for the WL group, with a mean of 3.28 kg (*P* < 0.01) for all participants losing weight as compared to an average body weight increase of 2.22 kg in the WG group. Further segmentation by gender among the weight loss group revealed that female participants lost 3.17 kg (*P* < 0.01) of body weight as compared to male participants at 4.06 kg weight loss (*P* < 0.01). Both of the WL values were significantly different from those in the WN and WG categories.

**Table 2:** Change in health measures, eating behaviors, and confidence values by weight loss category.

**Variable**	**Weight loss**	**Weight neutral**	**Weight gain**
Change of body weight, kg	3.28^**^	0.00	2.22
Females	3.17^**^	0.00	2.17
Males	4.06^**^	0.00	2.61
**Food intake (servings per day)**			
**High fiber food composite, servings per day**			
Baseline	6.26	6.58	6.22
Follow-up	9.07^**^	8.51	8.09
Change	2.81	1.93	1.87
**Fruit, servings per day (0–7)**			
Baseline	1.54	1.64	1.53
Follow-up	2.45^**^	2.28	2.13
Change	0.91	0.64	0.60
**Vegetables, servings per day (0–7)**			
Baseline	2.03	2.09	2.08
Follow-up	2.99^**^	2.73	2.63
Change	0.96	0.64	0.55
**Beans, servings per day (0–4)**			
Baseline	0.63	0.67	0.62
Follow-up	1.03^**^	0.98	0.87
Change	0.41	0.31	0.26
**Grains, servings per day (0–6)**			
Baseline	1.27	1.35	1.22
Follow-up	1.61^*^	1.57	1.50
Change	0.34	0.22	0.27
**Nuts, servings per day (0–4)**			
Baseline	0.80	0.84	0.77
Follow-up	1.00	0.97	0.95
Change	0.20	0.13	0.18
**Water intake**			
Baseline	5.73	5.93	5.83
Follow-up	7.08^**^	6.80	6.63
Change	1.35	0.87	0.80
**Health values (self-reported)**			
**Perceived confidence level for losing weight (0–10)**			
Baseline	5.16	5.03	5.14
Follow-up	6.45^**^	5.71	5.39
Change	1.29	0.68	0.24
**Perceived confidence level for choosing healthy food choices (0–10)**			
Baseline	6.10	6.11	6.03
Follow-up	7.46^**^	6.96	6.77
Change	1.37	0.85	0.74
**Perceived health value, 1–5**			
Baseline	3.14	3.25	3.23
Follow-up	3.41^**^	3.39	3.31
Change	0.27	0.14	0.08
**Perceived energy value, 0–6**			
Baseline	2.84	2.91	2.82
Follow-up	3.33^**^	3.21	3.10
Change	0.49	0.30	0.28

Significant differences between groups as determined by analysis of variance and post hoc follow-up tests (Tukey). Weight loss is the reference with comparison to weight neutrality and weight gain.

^**^P <0.01.

^*^P <0.05.

Perceived health value and water intake were only significantly different between WL and WG.

Previous studies demonstrated that FPL participants had statistically significant improvements in eating behaviors of fiber-rich foods between baseline and follow-up (20). Unique to this study was the development of a total fiber composite. As a result of the FPL program, participants in the WL group significantly increased their daily total intake of high-fiber foods by 2.81 servings to 9.07 daily servings (*P* < 0.01) as compared to 8.51 servings for WN and 8.09 servings for the WG group.

Daily servings of fruits, vegetables, beans, and grains were also significantly higher for the WL category at follow-up as compared to the WN and WG categories. The greatest change occurred in daily servings of fruits (0.91), vegetables (0.96), beans (0.41), and grains (0.34). The WL category recorded significantly higher servings of fruits, vegetables, and beans at 2.45 (*P* < 0.01), 2.99 (*P* < 0.01), and 1.03 (*P* < 0.01) as compared to the WN and WG groups. The WL group also had a significantly higher daily intake of whole grains at 1.61 servings compared to the WN and WG groups of 1.57 and 1.50 servings, respectively (*P* < 0.01). There was no significant difference among servings of nuts at follow-up across the body weight categories.

Confidence levels in losing weight were significantly higher for the WL group at 6.45 (*P* < 0.01) as compared to the categories of WN at 5.71 and WG at 5.39. The WL group confidence levels in making healthy choices were also significantly higher at 7.46 (*P* < 0.01) as compared to the categories of WN at 6.96 and WG at 6.77. The self-perceived health values for the WL group were significantly higher at 3.41 than the WG group at 3.31 but not of the WN group at 3.39. Self-perceived energy values were significantly different for the WL group at 3.33 (*P* < 0.01) as compared to the WN group at 3.21 and 3.10 for the WG group.

Table 3 shows the relationship between fiber intake and body weight through the development of two models. Model 1 demonstrates that a higher intake of total high-fiber foods (composite) at follow-up was associated with lower body weight (*P* < 0.01). Model 2 revealed that of the individual food categories of high-fiber foods (fruits, vegetables, beans, whole grains, and nuts), a higher intake of vegetables or fruits was associated with weight loss (*P* < 0.01). As a result of the multilevel modeling analysis, increases in the intake of nuts, beans, or grains were not associated with weight change during the study period (*P* > 0.15).

**Table 3:** Within-subject associations between intake of fiber-rich food composition, individuals' fiber-rich food categories, and body weight.

**Variable**	**Model 1**			**Model 2**		
	**Estimate**	**SE**	***P*** **-value**	**Estimate**	**SE**	***P*** **-value**
Water	−0.207	0.039	<0.01	−0.193	0.39	<0.01
Fiber composite	−0.352	0.034	<0.01			
Vegetables				−0.658	0.094	<0.01
Fruits				−0.355	0.101	<0.01
Nuts				−0.126	0.147	0.392
Beans				−0.230	0.161	0.153
Grains				−0.088	0.101	0.385
Time	−2.330	0.140	<0.01	−2.259	0.143	<0.01

## Discussion

Research has demonstrated that an intake of foods high in fiber is associated with a favorable impact on health status and body weight. This study adds to the body of literature examining the impact of a plant predominant fiber-rich eating approach and its relationship to weight loss in employer settings. More specifically, this study examines the relationship of dietary fiber among individuals participating in the FPL program. As demonstrated in previous studies, there is a direct relationship between the intake of dietary fiber and weight loss (10, 11, 14–17).

The initial finding confirms that the 16-week FPL program results in weight loss among the majority of participants, with 62.5% reporting weight loss. These results can help to address the adverse impact of obesity on health and productivity in the workplace and community by providing a low-cost healthy eating program (21). FPL promotes a healthy nutrition eating approach through a small-step adoption of improved eating habits through the gradual consumption of 75% of nutrients from plant-based whole foods while incorporating sustained adoption of healthy living and nutrition principles.

Further analysis evaluated the difference between three weight groups: those who lost weight, maintained weight, and gained weight. Of the WL group, there was a significant increase in total and individual servings of high-fiber foods including fruits, vegetables, beans, whole grains, and nuts. The WL group consumed 9.07 total daily servings, which was an increase of 0.56 over the WN group, and 0.98 serving more than the WG group. More specifically, the greatest intake of fiber-rich foods came from vegetables at 2.99 or 0.26 and 0.36 servings more than the WN and WG groups, respectively. As a result, our second finding demonstrated that as a result of participating in the FPL program, both the total intake of high-fiber foods, as well as select fiber-rich foods, play a significant role in body weight loss as higher daily servings resulted in greater weight loss. As with previous studies (28–30), individuals who consume higher amounts of vegetables and fruits have greater weight loss as these foods are high in fiber but lower in calories than other high-fiber foods such as beans, grains, and nuts.

The third finding of this study supports the hypothesis that dietary fiber is associated with weight loss. In our analysis, the change in fiber intake from baseline to 16 weeks was a consistent and strong predictor of weight loss. The association between increased fiber intake in the total fiber composite (Model 1) as well as and Model 2, the increased intake of vegetables or fruits, while controlling for water intake. Weight loss increases as total fiber intake, or more specifically fruit or vegetable intake increases.

Additional findings demonstrated that individuals within the WL group also had higher values of perceived energy and confidence values for losing weight and making healthier choices as compared to the other groups. Individuals who improve their health and lose weight have also been shown to have great self-efficacy (31).

There were notable limitations to the study. First, the study was 16 weeks in length and may not be reflective of long-term results. Second, as noted in previous FPL studies (20, 23, 24), self-reported survey data were used for analysis, inclusive of estimated servings of food in place of a daily food log and corresponding analysis. Third, demographic data were limited to age and gender, preventing stratification or adjustment of participant data based on race, ethnicity, and/or socioeconomic status. Finally, attrition bias is one of the major challenges in nutrition and weight management programs, consistent with this study. Future studies of the FPL program should include a longer-term program evaluation period with improved survey instruments, standardized weight measurement, comparison group, and analysis of exercise-related impact.

Despite these limitations, the study has implications for nutrition interventions. Specifically, FPL program interventions delivered at the workplace have been demonstrated to improve weight loss and therefore overall health. Due to these benefits, the FPL program may offer employers a strategy to improve both workplace productivity and healthcare costs. Programs such as FPL are also beneficial to be delivered in clinical and community settings as they may improve patient and community health. The FPL program has been found to improve overall eating habits, and for individuals who consume significant increases in fiber intake, there is corresponding weight loss. In summary, this study identified that the FPL program was effective in improving weight loss as a result of increased intake of high-fiber foods, including the consumption of total fiber and fruits and vegetables. In addition, improvements were significantly higher for confidence in losing weight, confidence in making healthy choices, and perceived health and energy values for the WL group. Future studies could focus on exploring dietary fiber intake and weight loss as part of a longer-term program in multiple settings. In addition, fiber's role in improving health through the measurement of additional clinical markers should be considered in future FPL studies.

## Data availability statement

The original contributions presented in the study are included in the article/supplementary material, further inquiries can be directed to the corresponding author.

## Ethics statement

Ethical review and approval was not required for the study on human participants in accordance with the local legislation and institutional requirements. The ethics committee waived the requirement of written informed consent for participation.

## Author contributions

RKK: conceptualization, methodology, design and formal analysis, writing, reviewing, editing, and ongoing communications. AH, JC, and RS: data collection, design analysis assistance, manuscript review and editing, and liaisons of the data and background literature. PP-F: ongoing review and edits to the manuscript. BWS: conceptualization, design analysis assistance, writing, reviewing, and editing. All authors contributed to the article and approved the submitted version.
